# Addressing Health Disparities in Chronic Kidney Disease

**DOI:** 10.3390/ijerph111212848

**Published:** 2014-12-11

**Authors:** Ta-Chien Chan, I.-Chun Fan, Michael Shi-Yung Liu, Ming-Daw Su, Po-Huang Chiang

**Affiliations:** 1 Research Center for Humanities and Social Sciences, Academia Sinica, Taipei City 115, Taiwan; E-Mails: dachianpig@gmail.com (T.-C.C.); mhfanbbc@ccvax.sinica.edu.tw (I.-C.F.); liumike@gate.sinica.edu.tw (M.S.-Y.L.); 2 The Institute of History and Philology, Academia Sinica, Taipei City 115, Taiwan; 3 Institute of Taiwan History, Academia Sinica, Taipei City 115, Taiwan; 4 Department of Bioenvironmental Systems Engineering, National Taiwan University, Taipei City 106, Taiwan; E-Mail: sumd@ntu.edu.tw; 5 Institute of Population Health Sciences, National Health Research Institutes, Miaoli County 350, Taiwan

**Keywords:** chronic kidney disease, health disparity, prevalence, spatial analysis, geographic information systems

## Abstract

According to the official health statistics, Taiwan has the highest prevalence of end stage renal disease (ESRD) in the world. Each year, around 60,000 ESRD patients in Taiwan consume 6% of the national insurance budget for dialysis treatment. The prevalence of chronic kidney disease (CKD) has been climbing during 2008–2012. However, the spatial disparities and clustering of CKD at the public health level have rarely been discussed. The aims of this study are to explore the possible population level risk factors and identify any clusters of CKD, using the national health insurance database. The results show that the ESRD prevalence in females is higher than that in males. ESRD medical expenditure constitutes 87% of total CKD medical expenditure. Pre-CKD and pre-ESRD disease management might slow the progression from CKD to ESRD. After applying ordinary least-squares regression, the percentages of high education status and the elderly in the townships are positively correlated with CKD prevalence. Geographically weighted regression and Local Moran’s I are used for identifying the clusters in southern Taiwan. The findings can be important evidence for earlier and targeted community interventions and reducing the health disparities of CKD.

## 1. Introduction

Chronic kidney disease is an emerging global health threat [1] and causes substantial economic burden for countries all over the world [2,3]. With the number of type 2 diabetes patients around the world expected to double in the next 25 years, the number of patients with chronic kidney disease (CKD) and end-stage renal disease (ESRD) will also surge in the following years [4]. Taiwan not only has suffered a high disease burden of CKD, but also has the highest prevalence and the third highest incidence of ESRD in the world as of 2011 [5]. According to a statistical report from the Taiwan Society of Nephrology (http://www.tsn.org.tw/UI/K/K008.aspx), the percentages of Stage 5 ESRD patients in health promotion outpatient visits who chose hemodialysis, peritoneal dialysis, and kidney transplant are 77.3%, 22.5%, and 0.2%, respectively. Thus, the medical expenditure on the dialysis treatment is high and continuously growing. In 2012, the dialysis treatments among ESRD patients in Taiwan cost around one billion U.S. dollars, which accounted for 5.89% of the total medical expenditure of national health insurance (http://goo.gl/AqeiCk, p7). To deal with this serious situation, screening of earlier-stage CKD patients and active intervention in pre-CKD and pre-ESRD disease management can slow the progression of early-stage CKD to ESRD [6]. However, the most important thing is to prevent the incidence of CKD. Thus, personal and environmental risk factors should be identified and prevented.

In previous studies, low socioeconomic status [7], history of hypertension, diabetes [8], use of non-prescribed Chinese herb medicine [9], analgesic use [10], and metabolic syndrome [11] were all identified as possible risk factors for developing CKD. All these risk factors are at the individual level, and will be beneficial for health education and clinical management. However, for the public health scope, how to reduce the incidence and prevalence in the communities is the first priority mission. Before that, we have to identify where the hotspots are after adjusting for possible socio-economic factors. There are several possible factors affecting CKD disparity including race, gender, socioeconomic status, and rural residence. Those factors would affect not only the occurrence of CKD, but also the prognosis of CKD and the medical treatment of CKD and ESRD [12,13,14]. 

In this study, we will focus on how health disparities affect the geographical distribution of CKD in Taiwan. In addition, the identified clustering townships with high CKD prevalence rates will be the suggested first priority intervention townships.

## 2. Methods

### 2.1. Data Collection

The nationwide CKD and ESRD national health insurance data were applied from the National Health Research Institute in Taiwan. The coverage of national health insurance in Taiwan exceeded 99% of the whole population in Taiwan [15]. When patients went to hospitals or clinics for outpatient, emergency room or inpatient service, the physician needed to make the diagnosis for that medical visit, and the diagnosis included the free text medical record and the corresponding International Classification of Diseases, Ninth Revision (ICD-9) code. Then, these ICD-9 codes were logged into the national health insurance database. The definition of CKD in this study used the ICD-9 codes 585 (*i.e.*, chronic kidney disease). However, the clinical stage of CKD from stage 1 to stage 5 was not recorded in the data. The ESRD patients could only be extracted by the specific “case type” variable with the value “05” which represented dialysis treatment. The ESRD patients need to regularly see their physician to monitor their clinical progression and begin the next course of the dialysis treatment. The dialysis for one treatment course lasts 30 days. After each medical visit (*i.e.*, seeing the physician), the ESRD patient can then arrange their time schedule for the dialysis treatment. Thus, the medical visits here mean that the patients need to see the physician, not the patients’ dialysis schedule. The studied period was from 2008 to 2012.

The demographic variables including townships’ population densities, townships’ percentage of aborigines, townships’ percentage of people with college or above education status, townships’ percentage of the elderly (≥65 years old) were downloaded and calculated from the Taiwan socio-economic database maintained by the Ministry of the Interior (http://segis.moi.gov.tw/). The city or county adult smoking rates were downloaded from the Ministry of Health and Welfare (www.mohw.gov.tw/cht/DOS/DisplayStatisticFile.aspx?d=12043&s=1). The number of hospitals and clinics in each township was calculated from the national health insurance database and further divided by each township’s area to get the density of hospitals and clinics. This study was approved by the institutional review board (IRB) of Academia Sinica (IRB#: AS-IRB-BM 13057).

### 2.2. Geographical Prevalence Estimation 

**Figure 1:**
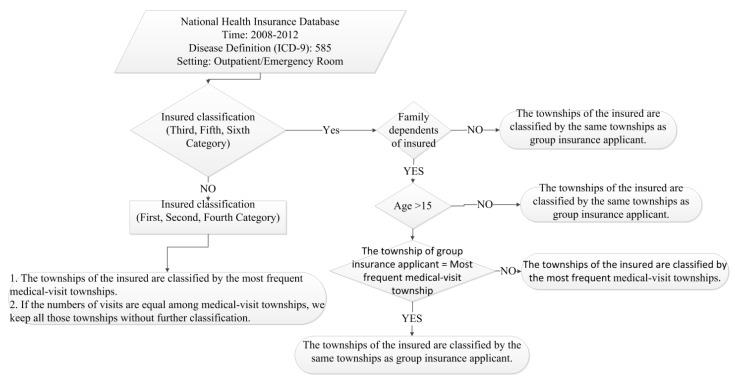
Flowchart estimating townships’ prevalence.

Although the comprehensive medical visits information is recorded in the national health insurance data, the townships of residence of the patients are unknown due to privacy concerns. This is a barrier to estimating local disease prevalence. However, we proposed one classification framework to estimate the patients’ location at the township level (Figure 1). In the data, we have two kinds of location information, which are the townships where the patients visited the hospitals or clinics, or the townships where the patients’ group insurance applicant is located. Before making this classification, we removed patients with CKD diagnosis but fewer than three CKD-related visits during 2008–2012, as they may not have been confirmed CKD cases. Such cases were not included for prevalence estimation, but were included in calculating the total CKD-related medical expenditure. 

According to Article 10 of the National Health Insurance Act in Taiwan (http://law.moj.gov.tw/Eng/LawClass/LawAll.aspx?PCode=L0060001), the insured classification has six categories based on the different types of job categories. Categories one, two and four were least likely to work in their township of residence. Categories three, five and six had higher tendency to work in their residential townships. Therefore, we firstly classified the patients by the insurance classification. A scrambled identification number was used for identifying each person with repeated medical visits. If the patients belonged to category one, two or four, we used the townships where their medical visits most frequently occurred as their townships of residence. If the numbers of visits were equal among two or more townships where medical visits were made, we keep all those original townships without further classification. If the patients belonged to category three, five or six, we assigned township of residence based on the following criteria. If the patients’ insured type was not family dependent, or was family dependent aged less than or equal to 15 years old, the township where the group insurance applicant was located was assigned. If the patients were aged greater than 15 years old and the most frequent medical-visit township was the same as the group insurance applicant’s township, the group insurance applicant’s township was assigned. For all other cases, the township where they made the most frequent medical visits was assigned. After making the classification, the prevalence rate was calculated by the number of CKD patients divided by the corresponding year’s population in the township.

### 2.3. Statistical Analysis

In examining the temporal trend of the CKD and ESRD medical visits and medical expenditures, the non-parametric statistical method, Jonckheere-Terpstra test, was applied with SPSS 20.0 (IBM Corp., Armonk, NY, USA). The comparison of medical visits and expenditures between males and females used an independent *t*-test.

In spatial analysis, we used global Moran’s I and local Moran’s I with ArcGIS (ArcMap, version 10.2; ESRI Inc., Redlands, CA, USA) for evaluating the spatial autocorrelation of CKD prevalence before Geographically Weighted Regression (GWR). Then, we used those two indicators again for evaluating the spatial autocorrelation of the residuals after GWR. A higher positive Moran’s I indicates that values in the neighboring areas tend to cluster, while a lower negative Moran’s I implies that higher and lower values are interspersed. When Moran’s I is close to 0, there is no spatial clustering, meaning that the data are randomly distributed [16]. A Local Moran’s I (LISA) cluster map of the residuals was created for identifying the clusters which cannot be explained by the current risk factors [17].

To understand how spatial disparity factors affect the CKD prevalence, we applied GWR to estimate and consider the spatial variability of each explanatory variable. The dependent variables we used here are the annual CKD prevalence in each township. The explanatory variables include population density, percentage of aborigines, percentage of education status (≥college), elderly percentage (≥65 years old), smoking rate and the density of hospitals or clinics.

Unlike conventional ordinary least-squares regression (OLS), GWR model is a type of local statistic in which the parameter estimations vary over space. The assumption of spatial non-stationarity is made in GWR, which means the correlations between the independent variable and dependent variables are not the same for every area [18]. We apply OLS analysis to observe the correlations between CKD and risk factors and do further GWR analysis by free software GWR 4.0 (http://www.st-andrews.ac.uk/geoinformatics/gwr/gwr-downloads/) [19]. The coefficients of each explanatory variable and the R-square in both OLS and GWR will be summarized in the results. The multi-collinearity is checked by variance inflation factor (VIF). If VIF is larger than 5, we will drop that variable for further OLS and GWR analysis. The residual maps after GWR and the LISA of the residuals will be displayed in the results with ArcGIS. 

## 3. Results

**Figure 2:**
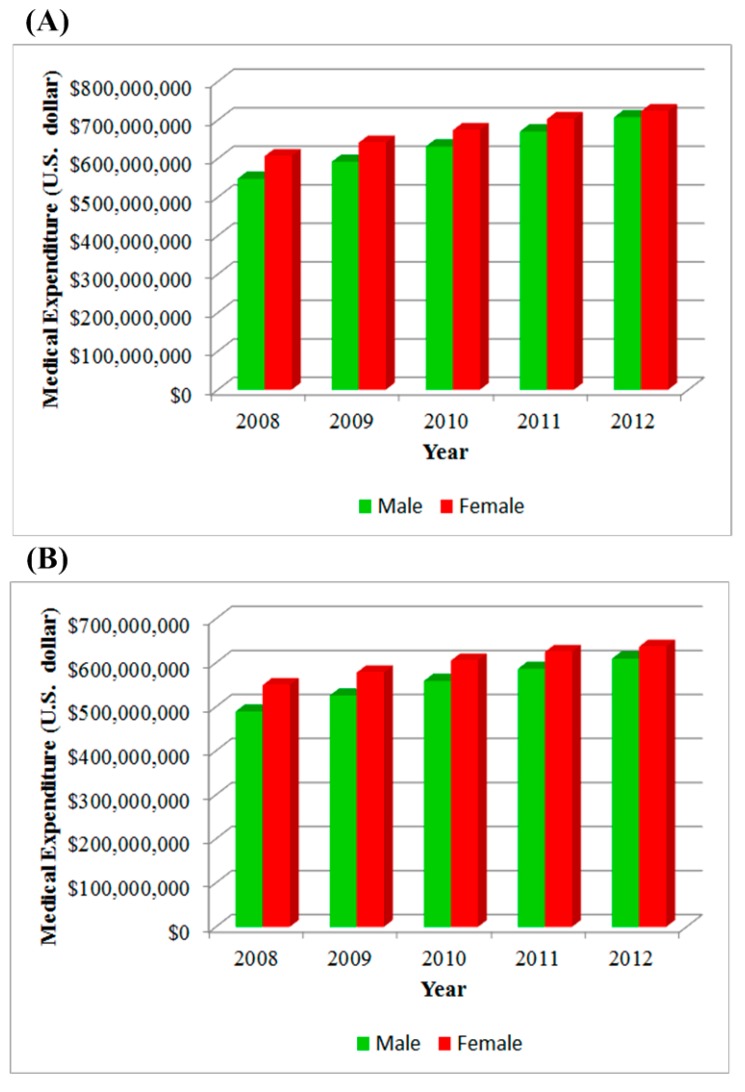
The medical expenditure paid by national health insurance: (**A**) chronic kidney disease; (**B**) end-stage renal disease.

The medical visits of CKD and ESRD by both genders significantly increased from 2008 to 2012 (Table 1, *p* = 0.01). The total CKD visits by males were higher than those by females (*p* = 0.5), but the ESRD visits by females were higher than those by males (*p* = 0.15). The total medical expenditures of CKD (*p* = 0.29) and ESRD (*p* = 0.13) paid by national health insurance were higher for females than males, and the expenditures continued increasing with time (Figure 2, *p* = 0.01). We assumed 1 reimbursed point value was equal to 1 New Taiwan Dollar (NTD) and the currency exchange rate in 2012 for U.S. dollars (USD) to NTD was about 1 to 30. In 2012, around 1.4 billion USD were spent on CKD medical treatment, including confirmed and suspected CKD patients; of this, 87% was for ESRD. 

**Table 1:** The medical visits of CKD and ESRD stratified by gender and year.

Year	CKD		ESRD	
	Male	Female	Male	Female
2008	1,090,712	1,058,147	308,493	347,169
2009	1,230,749	1,166,880	331,580	365,532
2010	1,374,800	1,273,005	352,702	383,122
2011	1,563,096	1,418,837	373,201	399,351
2012	1,833,066	1,605,235	392,413	411,323
Sum	7,092,423	6,522,104	1,758,389	1,906,497

**Figure 3:**
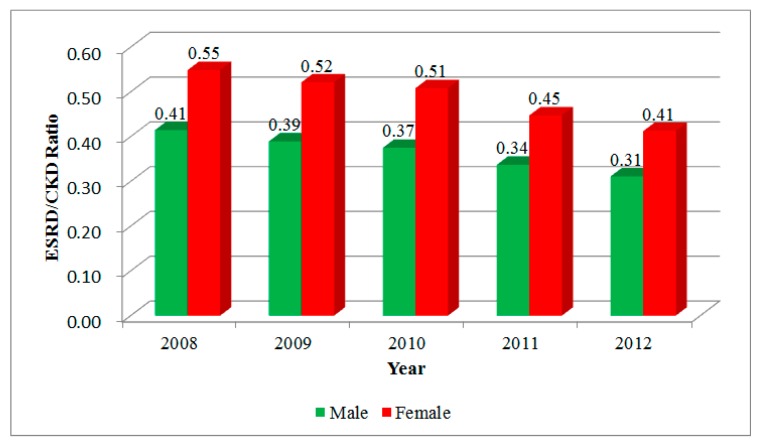
The ratios of ESRD and CKD.

The estimated prevalences of CKD and ESRD showed trends similar to those of medical visits. The prevalence of CKD in 2008 was 555.39, *versus* 892.45 in 2012 per 100,000 population (Table 2). The prevalence of ESRD in 2008 was 264.08, compared to 317.21 per 100,000 population in 2012. The major difference is the counting of persons instead of visits in the prevalence. In addition, we filter out suspected CKD cases in which the total number of CKD-related visits within the studied period is less than three. The ratios between ESRD and CKD for both genders are declining with time, which means the incidence of ESRD has slowed recently (Figure 3).

**Table 2:** The estimated prevalence of CKD and ESRD per one hundred thousand population.

Year	CKD			ESRD		
	Male	Female	Total	Male	Female	Total
2008	601.11	508.81	555.39	249.24	279.21	264.08
2009	682.72	559.28	621.41	265.85	291.60	278.64
2010	755.01	600.87	678.30	283.07	305.40	294.18
2011	886.97	704.36	795.93	298.84	315.18	306.99
2012	1003.01	781.59	892.45	312.15	322.28	317.21

**Figure 4:**
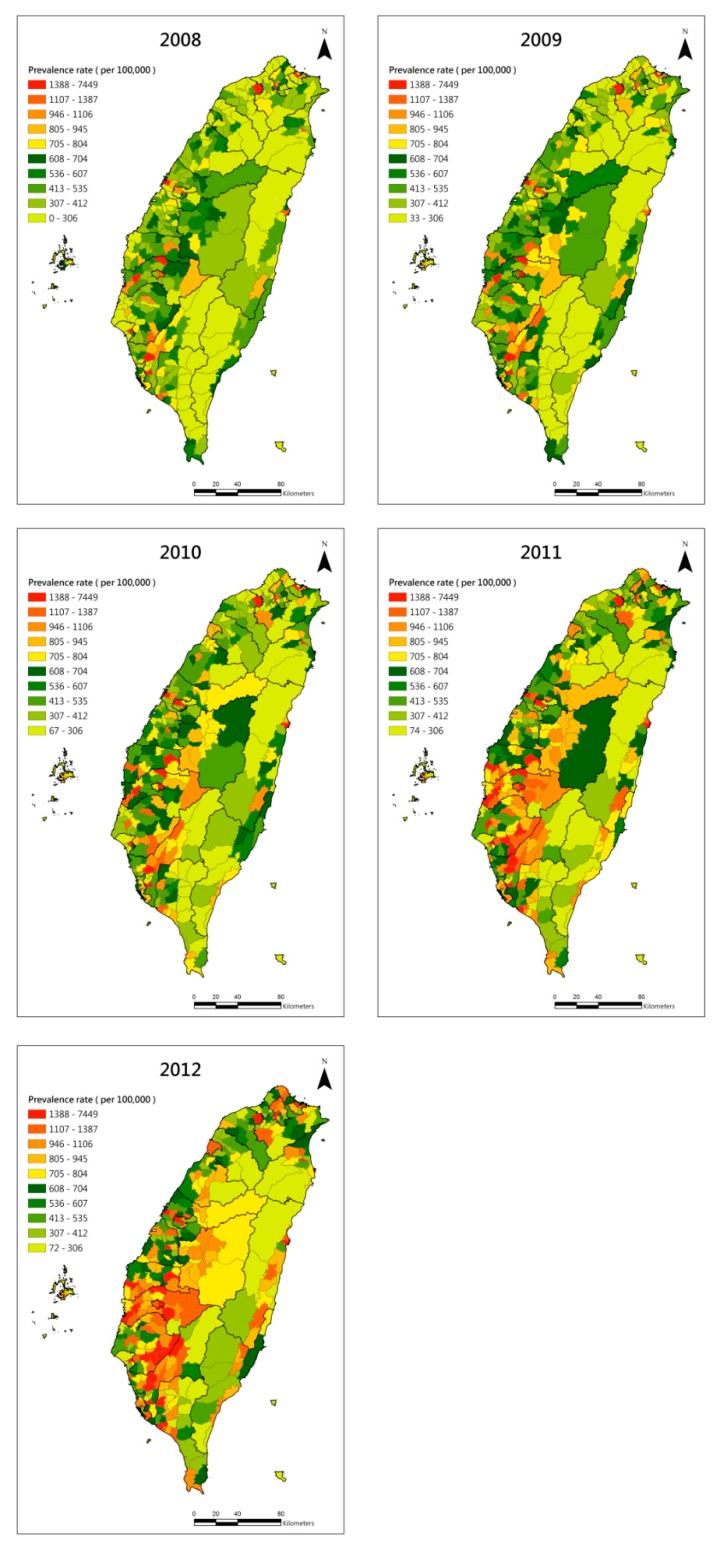
Geographical distribution of the estimated CKD prevalence in the township level of Taiwan from 2008 to 2012.

The geographical distribution of the estimated CKD prevalence each year is shown in Figure 4. The major clustering area was in southern Taiwan. To incorporate the possible disparity factors into the OLS model, we found that the townships with a higher percentage of the elderly and higher percentage of college or above education status had significantly higher CKD prevalence throughout the studied period (Table 3). In the exploratory analysis and correlation analysis, we found the VIF was high (VIF > 5) in both population density and density of clinics. Thus, we dropped the population density and kept density of clinics, which was directly correlated to the medical seeking behavior. In 2011 and 2012, we found the density of hospitals had significantly positive correlation with CKD prevalence, and the density of clinics had significantly negative correlation with CKD prevalence.

**Table 3:** The explanatory factors on CKD prevalence by ordinary least squares model.

Variables	2008			2009			2010			2011			2012		
	Beta	S.E.	VIF	Beta	S.E.	VIF	Beta	S.E.	VIF	Beta	S.E.	VIF	Beta	S.E.	VIF
% Aborigines	−1.2	27.6	1.8	5.0	31.5	1.9	8.7	35.9	1.9	7.8	43.3	1.9	24.3	47.9	1.9
% Residents aged >15 years with college or above education status	146.2 *	38.1	3.5	161.2 *	42.1	3.3	190.9 *	49.5	3.5	234.7 *	57.4	3.4	289.9 *	64.5	3.4
% Elderly aged ≥65 years	94.3 *	28.3	1.9	111.6 *	32.1	1.9	139.6 *	37.4	2.0	184.5 *	44.1	2.0	234.9 *	50.4	2.1
Smoking rate	−20.3	21.1	1.1	−32.1	24.3	1.1	−20.0	27.9	1.1	−13.9	32.1	1.1	−8.8	36.3	1.1
Density of hospitals	24.5	38.1	3.5	19.4	41.1	3.2	63.9	45.5	3.0	105.6 ^#^	53.6	3.0	211.6 *	58.9	2.8
Density of clinics	−49.8	44.2	4.7	−51.0	48.0	4.3	−91.6	53.3	4.1	−126.6 ^#^	62.7	4.1	−207.8 *	69.1	3.9
Adjusted R-square	0.08		0.08		0.08		0.09		0.12						

* *p* < 0.01; ^#^ *p* < 0.05.

**Figure 5:**
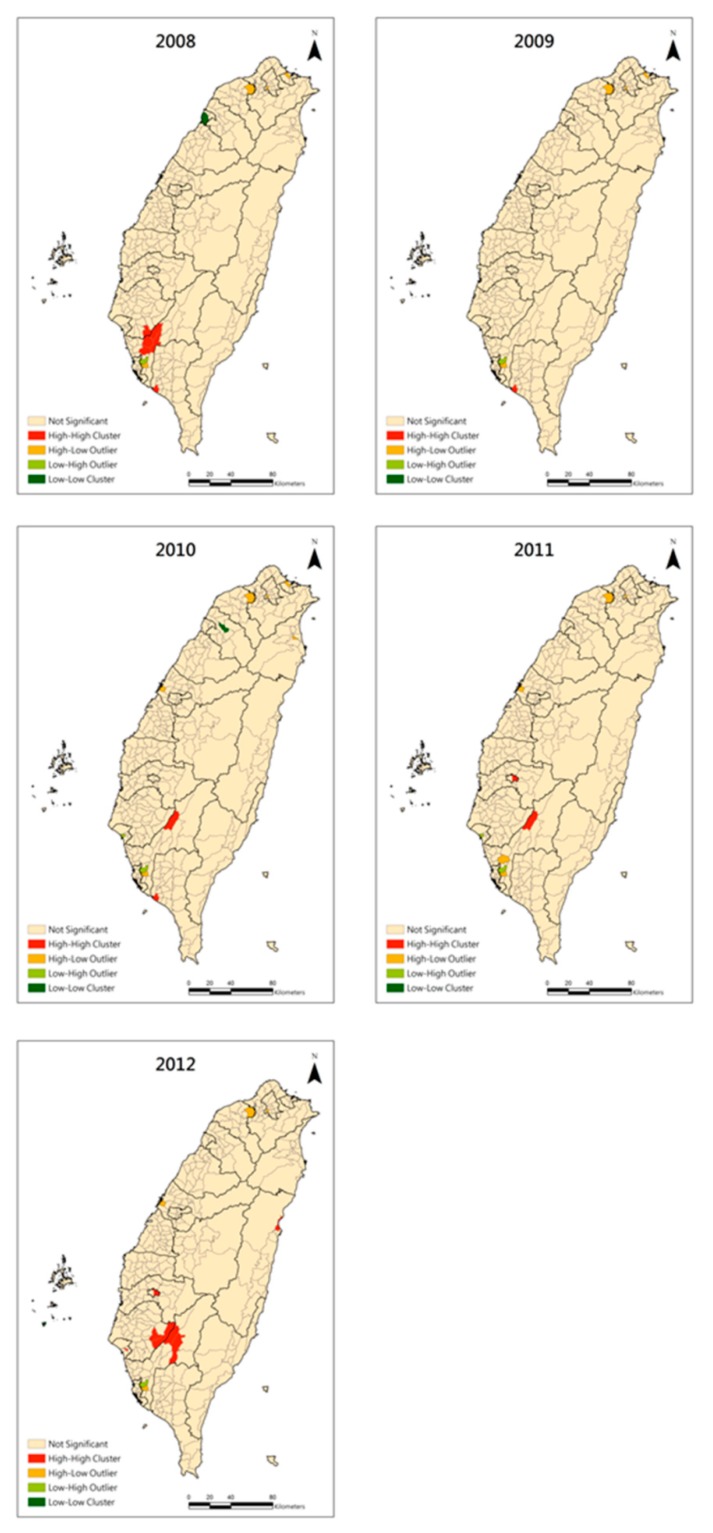
Local Moran’s I of the residuals by geographically weighted regression from 2008 to 2012.

**Figure A1:**
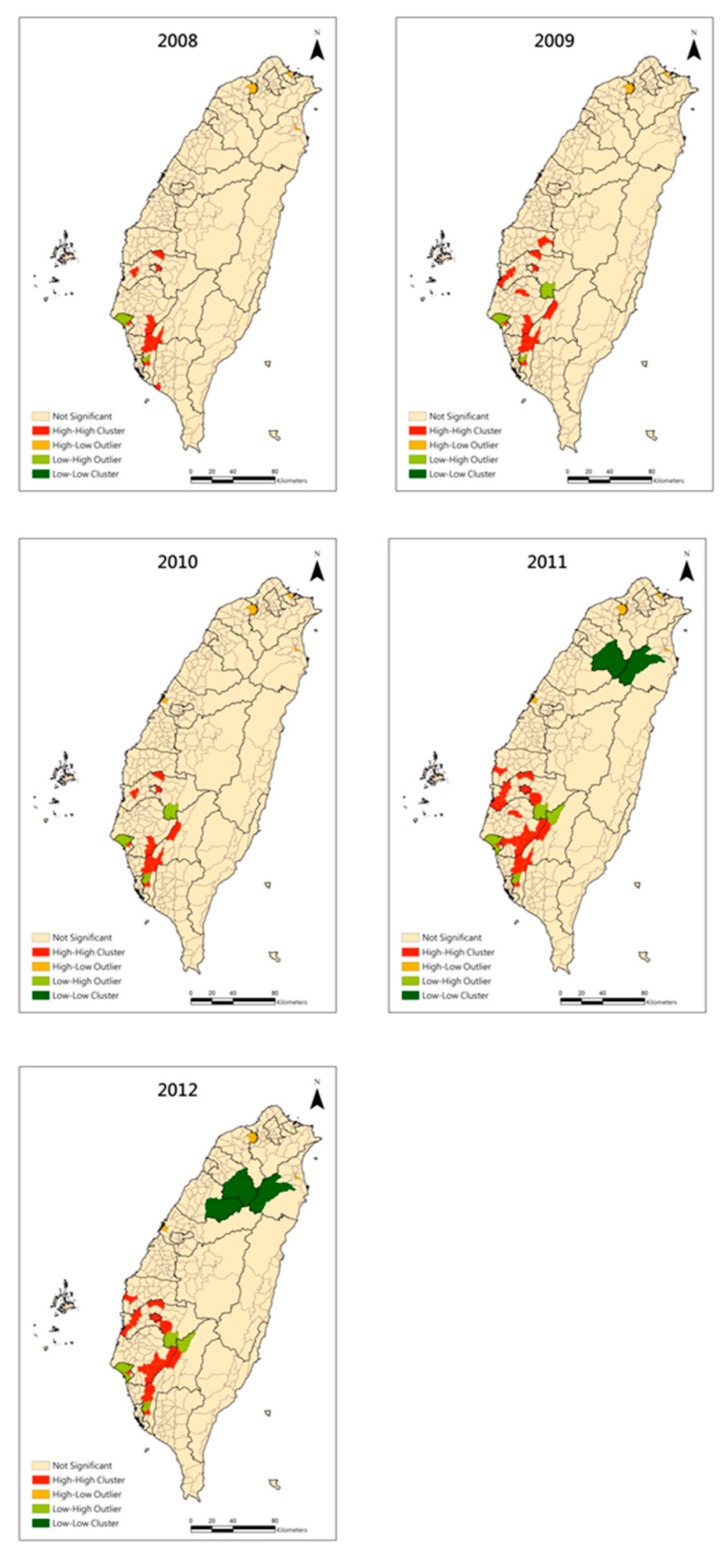
Local Moran’s I of the CKD prevalence from 2008 to 2012.

**Figure A2:**
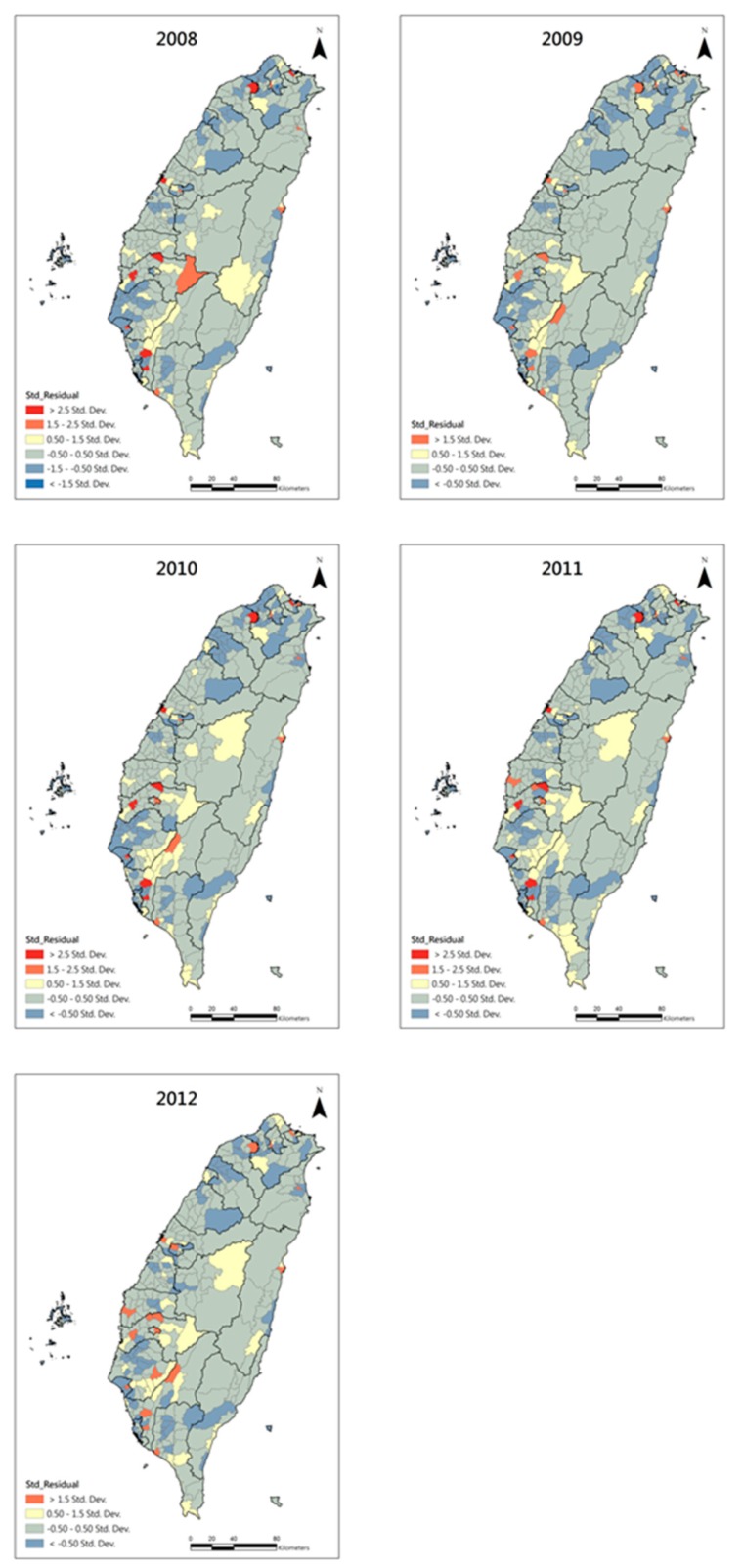
The residual maps after geographically weighted regression from 2008 to 2012.

Before we did GWR, we both used global Moran’s I and LISA for testing the spatial autocorrelation of CKD prevalence. In Table 4, we found that CKD prevalence was significantly clustered in 2011 and 2012 before GWR. Although the z-score of the Moran’s I was small, it was affected by the assumption of spatial homogeneity. Thus, we further applied LISA to examine the local clustering. In the map of LISA (Figure A1), we can clearly identify the high-high clusters in the five counties or cities of southern Taiwan. The residual map after GWR is shown in Figure A2, and the positive standard deviation of the residuals still showed major clustering in southern Taiwan and had few sporadic high residuals in northern and central Taiwan. To better identify the hot-spot clusters, the LISA map of the residuals is shown in Figure 5. The clear high-high clusters are within Chiayi City and the border townships between Tainan County and Kaohsiung County. 

**Table 4:** Examining the global spatial autocorrelation of CKD prevalence by Moran’s I before and after GWR from 2008 to 2012.

Year	Before GWR		After GWR	
	Moran’s I	*p*-Value	Moran’s I	*p*-Value
2008	0.01	0.20	−0.28	0.78
2009	0.01	0.24	−0.52	0.60
2010	0.01	0.33	−0.72	0.47
2011	0.02	0.01	−0.83	0.40
2012	0.03	0.00	−0.56	0.58

After GWR (Table 5), the overall adjusted R-square had 2%–8% improvement from the OLS model. Positive correlations were found for percentage of aborigines, the education status and the percentage of elderly based on the median coefficients among all townships. The smoking rate and the densities of hospitals, and clinics did not show a consistent pattern across the five years.

**Table 5:** The explanatory factors on CKD prevalence by geographically weighted regression.

Variables	2008		2009		2010		2011		2012	
	Median	R. STD	Median	R. STD	Median	R. STD	Median	R. STD	Median	R.STD
% Aborigines	17.0	27.5	26.7	32.7	33.5	45.0	39.8	67.3	68.9	97.1
% Residents aged >15 years old with college or above education status	169.6	114.2	191.0	125.1	233.2	153.7	294.7	319.6	349.8	354.5
% Elderly aged ≥65 years old	114.5	50.4	135.0	59.4	169.7	74.9	253.3	186.3	320.0	226.4
Smoking rate	−5.4	32.5	−29.8	19.0	−5.2	15.9	47.4	115.3	32.4	43.3
Density of hospitals	2.8	16.7	−5.7	20.4	36.7	13.1	6.2	167.1	−1.5	284.4
Density of clinics	−15.9	32.4	−16.1	34.9	−48.7	75.8	13.6	300.6	18.6	422.0
Adjusted R-square	0.11		0.10		0.11		0.16		0.20	

Note: R. STD = Robust standard error (interquartile range/1.349).

## 4. Discussion and Conclusions 

In this study, we firstly analyze the latest five years’ temporal trend of CKD medical utilization and expenditure in Taiwan. We have found that CKD and ESRD caused high medical expenditure each year, and the total expenditure has gradually risen over time. Other countries such as Australia [20] and the United Kingdom [21] have also faced increasing and tremendous medical expenditures on CKD. In Taiwan, there are many factors to explain such high medical expenditures. The first is the 99% national health insurance coverage of the whole population. This reduces the economic barrier to medical visits for the public, and has caused a substantial reduction in deaths after national health insurance (NHI) was implemented in 1995 [15]. However, NHI also stimulated the growth of the population being treated for ESRD, due to the affordable medical cost [22]. 

The second factor is the trend of an increasing aging population with diabetes, hypertriglyceridemia, and hyperuricemia [23,24]. Non-elderly adults also have an increasing trend of CKD, which could be correlated to lifestyle and metabolic syndrome [25]. Although CKD prevalence among males is higher than among females, ESRD prevalence is higher in females. One previous study found that male pre-ESRD patients had better CKD resilience than females [26]. That might be due to a difference in health-promoting behaviors, causing different progession of the disease, which would also explain why the total CKD expenditure for females is higher than that for males, due to the higher percentage of female ESRD patients. The average age of female CKD patients was a little bit older than male CKD patients in this study (male: 64.3 (SD: 14.2) ; female: 65.1 (SD: 13.6)), but did not reach the significance level. It is worth figuring out the age of the first dialysis and the onset age of CKD patients in both genders with cohort data. In addition, the competing risk of other chronic diseases and higher mortality in male CKD patients before developing ESRD might also be a possible reason for the lower ESRD prevalence among males. But our data cannot address this question adequately. 

In Taiwan, the pre-ESRD intervention started in November 2006, and pre-CKD intervention in 2011. U.S. Renal Data System report [27] showed that ESRD incidence in Taiwan was 384 per million population in 2003, and in 2011 it was 361 per million population. The trend of ESRD incidence was indeed declining. Thus, we can see the ESRD prevalence in this study also reflected this pattern. However, the prevalence of CKD increased rapidly, and pre-CKD and pre-ESRD disease management was also implemented during this stage. Thus, we might not clearly differentiate these two factors as reasons for the declining trend of the ratio of ESRD to CKD. In another study, good effectiveness of pre-CKD and pre-ESRD disease management in slowing ESRD incidence recently has been observed [6]. 

In the OLS results, we found the townships with a higher percentage of education status or higher percentage of elderly had higher CKD prevalence. These results show two different patterns of townships. The first is correlated with the metropolitan cities which have more than 30% metabolic syndrome prevalence among those aged 40 years and over [28]. The second is correlated with the traditionally agricultural or aboriginal townships, especially those with higher CKD incidence in southern Taiwan [8]. After GWR analysis and a clustering test, we find that in southern Taiwan, we indeed can identify a few higher CKD prevalence townships. It would be worth investigating any local risk factors among those high-high clustering townships in the future. 

**Figure A3:**
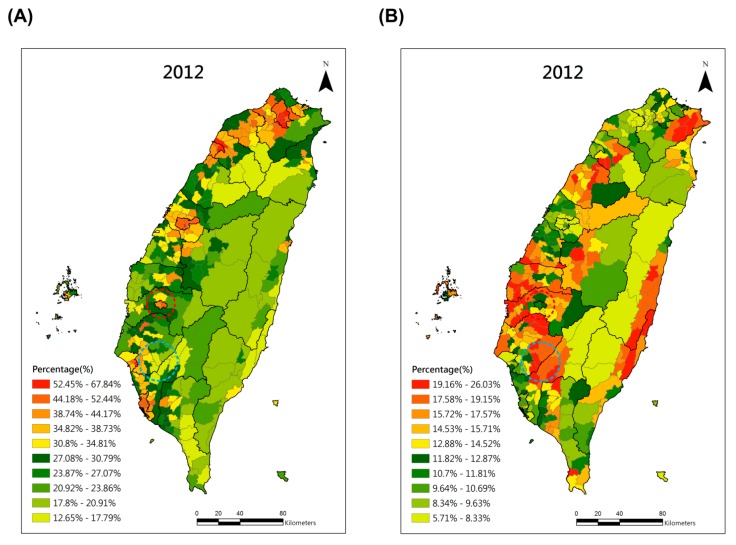
Significant explanatory variables on CKD prevalence in 2012 (**A**) Percentage of residents aged > 15 years old with college or above education status; (**B**) Percentage of elderly aged ≥ 65 years old.

Note: Red circle represents Chiayi city and the blue circle represents the border of Tainan county and Kaohsiung county.

Although the overall trend found the educational level was positively correlated with the CKD prevalence, we identified some high-high clustering townships with low educational level in the border townships between Tainan County and Kaohsiung County by GWR and LISA. The findings are consistent with the other published studies [29,30]. However, we also found some hotspot areas in Chiayi City identified by the residual maps which had high educational level. The spatial distribution of education level and the elderly in 2012 are shown in Figure A3. The risk factors of CKD in urban and rural townships might be different. In urban townships, the increasing trend of metabolic syndrome and diabetes might be correlated with the CKD incidence. Education level, which means the percentage of residents aged >15 years old with college or above education status, served as the proxy indicator of average income level for the townships. The economic barrier for medical visits or dialysis treatment is quite low in Taiwan because most of the medical expenditure is covered by the national health insurance. However, the accessibility was quite different between urban and rural townships. Thus, we can find that CKD prevalence was positively correlated with density of hospitals and negatively correlated with density of clinics from OLS results. This reflects the fact that access to medical resources would affect the patients’ medical seeking behavior. The medical resources were high in the urban townships, and those townships usually had a higher percentage of people with high educational level. The other observation is that non-elderly adults also have an increasing trend of CKD, which could be correlated to lifestyle and metabolic syndrome [25]. Therefore, overall, urban or populated areas with higher education levels tend to have higher CKD prevalence.

However, there are still many limitations in this study. The first one is the estimation of the patients’ locations. As we mentioned in the methods, the patients’ real locations are not available due to privacy concerns. Thus, we have to use limited information on insured classification, locations of group insurance applicant and most frequent medical visits to estimate their locations. However, the location of some people could not be estimated with NHI data. The other issue is that if any patients prefer visiting hospitals far from both their home and work place, we also cannot estimate their townships of residence correctly. 

The second limitation is the confirmation of CKD patients. We used three or more CKD-related medical visits during the entire follow-up period as the criterion for CKD patients for further examination, in order to rule out some patients with merely suspected CKD. The definition of the CKD patient here was not based on clinical confirmation, and a high estimation of CKD prevalence might have occurred. 

The third limitation is the lack information on the prevalence of regular intake of folk remedies or over-the-counter Chinese herbs. This might be the reason for some hotspot townships, but evidence is still needed to validate this. 

To our knowledge, this is the first paper to estimate spatio-temporal CKD prevalence at the township level and identify possible hotspots of CKD prevalence in Taiwan. With this study, we found that there were more female than male ESRD patients. Health education and clinical management should thus pay attention more to early female CKD patients. The pre-CKD and pre-ESRD disease management might have effects in slowing the progression from CKD to ESRD. The CKD prevalence in southern Taiwan is persistently high over time. Targeted intervention and epidemiological investigation are urgently needed. 

## References

[1] Perico N., Remuzzi G. (2012). Chronic kidney disease: A research and public health priority. Nephrol. Dial. Transplant..

[2] Jha V., Wang A.Y., Wang H. (2012). The impact of CKD identification in large countries: The burden of illness. Nephrol. Dial. Transplant..

[3] Nugent R.A., Fathima S.F., Feigl A.B., Chyung D. (2011). The burden of chronic kidney disease on developing nations: A 21st century challenge in global health. Nephron. Clin. Pract..

[4] Atkins R.C. (2005). The epidemiology of chronic kidney disease. Kidney Int..

[5] United States Renal Data System (USRDS) (2013). 2013 Annual Data Report: Atlas of Chronic Kidney Disease and End-Stage Renal Disease in the United States.

[6] Lin C.M., Yang M.C., Hwang S.J., Sung J.M. (2013). Progression of stages 3b-5 chronic kidney disease—Preliminary results of Taiwan national pre-ESRD disease management program in Southern Taiwan. J. Formos. Med. Assoc..

[7] Tsai S.Y., Tseng H.F., Tan H.F., Chien Y.S., Chang C.C. (2008). End-stage renal disease in Taiwan: A case-control study. J. Epidemiol..

[8] Kuo H.W., Tsai S.S., Tiao M.M., Yang C.Y. (2007). Epidemiological features of CKD in Taiwan. Am. J. Kidney Dis..

[9] Hsieh C.F., Huang S.L., Chen C.L., Chen W.T., Chang H.C., Wu M.L., Yang C.C. (2012). Increased risk of chronic kidney disease among users of non-prescribed Chinese herbal medicine in Taiwan. Prev. Med..

[10] Hsu Y.C., Lee P.H., Lei C.C., Shih Y.H., Lin C.L. (2014). Analgesic use, parents’ clan, and coffee intake are three independent risk factors of chronic kidney disease in middle and elderly-aged population: A community-based study. Renal Fail..

[11] Singh A.K., Kari J.A. (2013). Metabolic syndrome and chronic kidney disease. Curr. Opin. Nephrol. Hypertens..

[12] Plantinga L., Howard V.J., Judd S., Muntner P., Tanner R., Rizk D., Lackland D.T., Warnock D.G., Howard G., McClellan W.M. (2013). Association of duration of residence in the southeastern United States with chronic kidney disease may differ by race: The Reasons for Geographic and Racial Differences in Stroke (REGARDS) cohort study. Int. J. Health Geogr..

[13] Norris K., Nissenson A.R. (2008). Race, gender, and socioeconomic disparities in CKD in the United States. J. Am. Soc. Nephrol..

[14] Rodriguez R.A., Hotchkiss J.R., O’Hare A.M. (2013). Geographic information systems and chronic kidney disease: Racial disparities, rural residence and forecasting. J. Nephrol..

[15] Lee Y.C., Huang Y.T., Tsai Y.W., Huang S.M., Kuo K.N., McKee M., Nolte E. (2010). The impact of universal National Health Insurance on population health: The experience of Taiwan. BMC Health Serv. Res..

[16] Ta-Chien Chan, King C.-C., Castillo-Chavez H.C., Lober W.B., Thurmond M., Zeng D. (2010). Surveillance and Epidemiology of Infectious Diseases Using Spatial and Temporal Clustering Methods. Infectious Disease Informatics and Biosurveillance: Research, Systems and Case Studies.

[17] Chan T.C., Chiang P.H., Su M.D., Wang H.W., Liu M.S. (2014). Geographic disparity in chronic obstructive pulmonary disease (COPD) mortality rates among the Taiwan population. PLoS One.

[18] Fotheringham A.S., Brunsdon C. (1999). Local forms of spatial analysis. Geogr. Anal..

[19] Nakaya T., Fotheringham A.S., Brunsdon C., Charlton M. (2005). Geographically weighted Poisson regression for disease association mapping. Stat. Med..

[20] Tucker P.S., Kingsley M.I., Morton R.H., Scanlan A.T., Dalbo V.J. (2014). The increasing financial impact of chronic kidney disease in Australia. Int. J. Nephrol..

[21] Kerr M., Bray B., Medcalf J., O’Donoghue D.J., Matthews B. (2012). Estimating the financial cost of chronic kidney disease to the NHS in England. Nephrol. Dial. Transplant..

[22] Yang W.C., Hwang S.J. (2008). Taiwan Society of Nephrology. Incidence, prevalence and mortality trends of dialysis end-stage renal disease in Taiwan from 1990 to 2001: The impact of national health insurance. Nephrol. Dial. Transplant..

[23] Kao Y.M., Chen J.D. (2013). Inverse association between body mass index and chronic kidney disease in older diabetic adults. Ann. Epidemiol..

[24] Lin M.Y., Chiu Y.W., Lee C.H., Yu H.Y., Chen H.C., Wu M.T., Hwang S.J. (2013). Factors associated with CKD in the elderly and nonelderly population. Clin. J. Am. Soc. Nephrol..

[25] Sun F., Tao Q., Zhan S. (2010). Metabolic syndrome and the development of chronic kidney disease among 118,924 non-diabetic Taiwanese in a retrospective cohort. Nephrology (Carlton).

[26] Ma L.C., Chang H.J., Liu Y.M., Hsieh H.L., Lo L., Lin M.Y., Lu K.C. (2013). The relationship between health-promoting behaviors and resilience in patients with chronic kidney disease. Sci. World J..

[27] United States Renal Data System (USRDS) USRDS Archived Annual Data Report: Atlas of End-Stage Renal Disease in the United States. National Institutes of Health, National Institute of Diabetes and Digestive and Kidney Diseases. http://www.usrds.org/archive.aspx.

[28] Lin C.C., Liu C.S., Lai M.M., Li C.I., Chen C.C., Chang P.C., Lin W.Y., Lee Y.D., Lin T., Li T.C. (2007). Metabolic syndrome in a Taiwanese metropolitan adult population. BMC Public Health.

[29] Bello A.K., Peters J., Rigby J., Rahman A.A., El Nahas M. (2008). Socioeconomic status and chronic kidney disease at presentation to a renal service in the United Kingdom. Clin. J. Am. Soc. Nephrol..

[30] Fored C.M., Ejerblad E., Fryzek J.P., Lambe M., Lindblad P., Nyren O., Elinder C.G. (2003). Socio-economic status and chronic renal failure: A population-based case-control study in Sweden. Nephrol. Dial. Transplant..

